# Application of 25 MHz B-Scan Ultrasonography to Determine the Integrity of the Posterior Capsule in Posterior Polar Cataract

**DOI:** 10.1155/2018/9635289

**Published:** 2018-03-26

**Authors:** Yawen Guo, Chengzhe Lu, Bin Wu, Jianmin Gao, Jun Li, Xiaoyong Yuan, Xin Tang

**Affiliations:** Tianjin Eye Hospital, Tianjin Key Lab of Ophthalmology and Visual Science, Clinical College of Ophthalmology, Tianjin Medical University, Tianjin, China

## Abstract

**Purpose:**

To report the application of 25 MHz B-scan ultrasonography (MHzB) to determine the integrity of the posterior capsule (PC) in posterior polar cataract (PPC).

**Methods:**

Patients with whom PPC was clinically diagnosed using slit lamp microscopy who underwent 25 MHzB before phacoemulsification were retrospectively reviewed. The status of the PC was determined by 25 MHzB before phacoemulsification and confirmed during cataract surgery.

**Results:**

In total, 21 eyes in 14 clinically diagnosed PPC patients were enrolled in this study. Out of 25 MHzB images, 19 PCs were found to be intact, while 2 showed dehiscence before cataract surgery. During phacoemulsification, 17 PCs were observed to be intact, while 4 PCs showed posterior capsule rupture (PCR). These 4 PCR cases included the above 2 eyes, in which preexisting dehiscence was detected by 25 MHzB. The other 2 PCR cases showed high reflectivity between high echoes in posterior opacities and the PC, indicating synechia between the PPC and PC.

**Conclusion:**

This is the first report to show that 25 MHzB can be used to clearly visualize the status of the PC in PPC. These results, in turn, could be used to select the appropriate treatment and to thereby avoid further complications during PPC surgery.

## 1. Introduction

Posterior polar cataract (PPC) is one of the most challenging types of cataract, and it has a high risk of intraoperative posterior capsule rupture (PCR) or even nuclear or cortical drop [1–4]. In addition, the diagnosis of these conditions depends mainly on clinical observations made under slit lamp biomicroscopy, and the following three clinical categories are based on these clinical presentations: PPC with intact posterior capsule (PC), PPC with preexisting PC dehiscence, and spontaneous dislocation [5, 6]. The risk of PCR is higher during cataract surgery for PPC partially as a result of the tight adherence of the posterior polar opacity and the reduced integrity of the thin PC [7–9].

Studies that explore preoperative evaluations of the integrity of the PC and the relationship between posterior opacity and the PC are very helpful for preparing for scheduling and designing surgical approaches. A diagnosis of PPC depends mainly on clinical manifestations, but only a few instruments can be used to evaluate the integrity of the PC before cataract surgery. Anterior segment optical coherence tomography (OCT) has been successfully used to identify eyes with PPC [7, 10]. In a relatively dense cataract with PPC, using these measurements provides information of only limited value in PPC. Performing routine B-Scan ultrasonography at 10 MHz does not detect the PC because of its low resolution, and while performing ultrasound biomicroscopy (UBM) at 35–50 MHz is useful for detecting the anterior segment of the eyeball, including the ciliary body, it does not reach the plane of the PC because of its lower penetration.

The MD-2300S A/B scanner that is currently available in Meda, Tianjin, can probe at 25 MHz according to ophthalmic requirement in addition to the general level of 10 MHz. This higher level provides clear, high-resolution images of PPC, which are valuable for evaluating patients in preparation for scheduled PPC surgery.

## 2. Patients and Methods

This study was approved by the ethics committee of the Tianjin Eye Hospital, and all procedures were performed in accordance with the Declaration of Helsinki. From July 2016 to June 2017, patients with a diagnosis of PPC made based on slit lamp microscopy who underwent 25 MHz B-scan ultrasonography (MHzB) before phacoemulsification were retrospectively reviewed to obtain data including the patients' demographics, preoperative and postoperative visual acuity, PC images at 25 MHzB, and the integrity of the PC during surgery. In addition, some patients with posterior subcapsular cataract who were examined with 25 MHzB to determine their PC status at the same time as the experimental group served as controls.

The 25 MHzB scanner is a customized product produced by Meda (Tianjin, China) that has a transverse resolution ≦ 0.2 mm, longitudinal resolution ≦ 0.15 mm, and penetrating power ≧ 20 mm. This device can be connected to a conventional 10 MHzB probe socket.

In all cases in this study, 25 MHzB was performed by the same experienced technician based on the protocol for PPC used at Tianjin Eye Hospital. All patients were situated in a supine position before the eye cup was positioned. Balanced salt solution (BSS) was then applied, and images were obtained with a specialized probe at 25 MHzB. The probe was immersed in BSS and focused on the PC. In addition, the images of the PPC were then analyzed and evaluated by two ophthalmologists before the scheduled phacoemulsification.

In the PPC cases, phacoemulsification was performed by the same experienced surgeons after a 5 mm capsulorhexis and hydrodelineation were performed. The phaco chop technique was used for the lens nucleus, leaving the posterior epinucleus, and the viscoelastic was injected carefully between PPC and PC before irrigation and aspiration of posterior epinucleus and cortex. A status of the PC was confirmed, and an intraocular lens (IOL) was inserted into the capsular bag in cases with an intact PC, whereas a sulcus IOL was placed in cases with PCR after an anterior vitrectomy was performed, if necessary.

## 3. Results

In all, 21 eyes in 14 patients with clinically diagnosed PPC were enrolled in this study.  Table 1 shows the clinical data in these patients. In all PPC cases, 25 MHzB was successfully performed to detect the configuration of the PC and the relationship between the PPC and PC.

**Figure 1:**
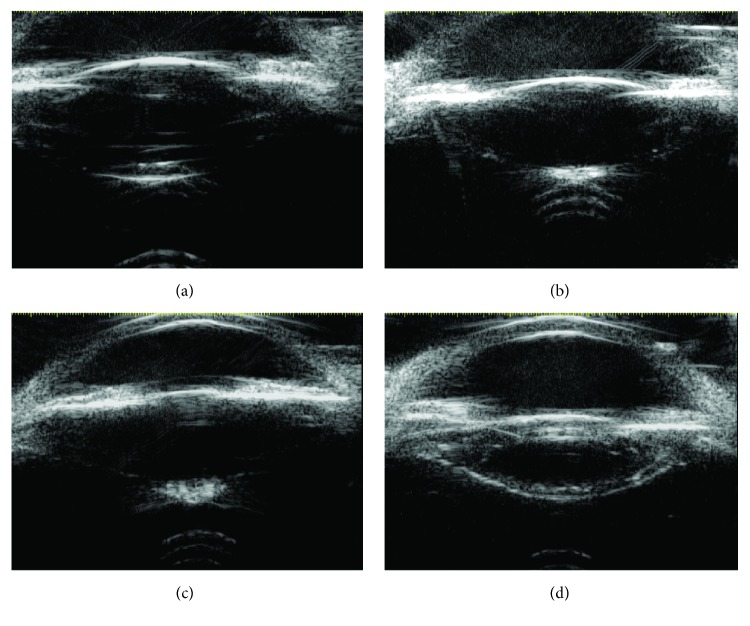
Images of the posterior capsule (PC) under 25 MHz B-scan ultrasonography (MHzB). (a) Normal continuous PC curve (CPCC) with minimal reflectivity between the PC and posterior polar cataract (PPC) in a clinically diagnosed case of PPC. (b) Normal CPCC with high reflectivity between the PC and PPC in a clinically diagnosed case of PPC. (c) PC dehiscence with reflectivity protruding into the vitreous cavity in a clinically diagnosed case of PPC. (d) Posterior subcapsular cataract with an intact PC.

Two categories of reflectivity were observed under 25 MHzB (Figure 1). Nineteen eyes showed a normal continuous PC curve (CPCC) with some extent of high reflectivity between a posterior opacity with high echoes and the PC, suggesting that the posterior polar cataract was located in front of the PC and had some extent of adhesion to the PC and that there was no congenital defect in the PC. During phacoemulsification, the PC was intact and there was some adhesion between the PPC and PC in 17 cases. PCR occurred during surgery in 2 of these cases, and after careful anterior vitrectomy was performed, the IOL was successfully placed into sulcus. In the other 2 cases, the PC exhibited dehiscence with reflectivity and protruded into the vitreous cavity when observed with 25 MHzB. In addition, PCR occurred in both of these cases during phacoemulsification. After the nucleus and cortex were carefully removed with anterior vitrectomy, the IOL was inserted into the sulcus (Table 2).

In all cases with clinically diagnosed PPC, hydrodelineation was performed during phacoemulsification. PCR was observed only after the nucleus was removed. In addition, no posterior nuclear drop occurred even in the cases with PCR. In addition, an IOL was inserted into the capsular bag in cases with an intact PC. Otherwise, it was inserted into the sulcus.

## 4. Discussion

Posterior polar cataract is a rare and special type of cataract that is characterized by white, calyx, concentric, or disc-like opacity in the center of the PC. Because the PPC is close to the optical nodal point of the eye, this condition usually affects visual quality. In addition, PCR with a dropped nucleus and cortex is more common than a normal cataract when dysplasia of the PC occurs. It is very important to anticipate the status of the PC to decrease the likelihood of severe complications during phacoemulsification for PPC [11–13]. While in PPC, diagnoses are mainly achieved by visualizing the posterior opacification under a slit lamp microscope; it is difficult to distinguish the relationship between the PPC and PC. This is especially dangerous in cases of congenital PC dehiscence for cataract surgeries [14–16].

Additionally, it is very difficult to evaluate the integrity of the PC before a cataract surgery because the plaque obstructs examination by all optical instruments that are normally used to measure the relationship between the PPC and PC. In the first type of PPC, the PC itself is intact, while in the second, the PC has a preexisting dehiscence [4, 5]. It is almost impossible to distinguish between the two types in clinical examinations performed using currently available instruments.

A routine B-scan examination does not provide high-enough resolution to scan the PC of the lens, and UBM signals do not reach the level of the PC because of their high frequency. It was previously reported that a pentacam or an anterior segment OCT could partially detect the relationship between the PC and PPC, but the results depend on the optical characteristics of the detection method, which limits the value of this new and promising technique for measuring PPC in dense cases or in those with a large area of PPC. While ultrasound is not affected by opaque soft tissues, the 25 MHzB could still detect the PC status even behind the dense opacity in the lens.

In 25 MHzB, the probe is attached to an MD-2300S A/B scanner (Meda Medical Science & Technical Ltd., Tianjin) that has special features, such as a vertical resolution ≦ 0.2 mm, lateral resolution ≦ 0.15 mm, and a penetrating resolution ≧ 20 mm. Under 25 MHzB, which is capable of focusing on the plane of the PC, the status of the PC can easily be detected, as can the relationship between the PPC and PC. This device uses ultrasound to detect the tissues, and ultrasound is not affected by opaque soft tissues, including PPC. The 25 MHzB therefore has an advantage over other currently available instruments for detecting PC status. In our current case series, 19 PC were found to be intact and 2 exhibited dehiscence. These latter cases were confirmed under 25 MHzB following phacoemulsification. In addition, another 2 cases of intact PC were detected under 25 MHzB and these exhibited normal CPCC and high reflectivity between the PC and PPC. Special care should be taken in these cases during phacoemulsification because this condition is associated with a high risk of PCR. The results of our study show that the 25 MHzB achieved very high accuracy for detecting defects in the PC in clinically diagnosed PPC.

Vasavada et al. [5] suggested that increasing our understanding abnormalities that are observed in the PC could decrease the incidence of PCR. The risk of PCR during phacoemulsification has decreased from 36% to 6-7% [1, 2, 13, 15, 17], a change that is partially explained by the success of different surgical techniques or modifications to them [7, 18], such as performing hydrodelineation only in cases without hydrodissection, viscodissection without nuclear rotation, inside-out delineation, slow-motion phacoemulsification, and layer-by-layer lens removal [19, 20]. All of these modifications are aimed at reducing stress on the PC to prevent PCR during cataract surgery.

In our case series, the status of the PC was predicted before phacoemulsification and the corresponding treatment was performed during surgery to prevent severe complications, such as hydrodelineation with a small amount of BBS instead of hydrodissection or leaving some of the epinucleus until the nucleus has been completely removed. As a result, even in the cases diagnosed with dehiscence on 25 MHzB in our study, hydrodelineation was performed as mentioned above and no dropping of the nucleus or cortex occurred when using this technique.

## 5. Conclusion

To our knowledge, this is the first report to describe the use of 25 MHzB to visualize the status of a PPC. Prejudging the status of the PC is vital to treating PPC, and 25 MHzB allows the practitioner to visualize the relationship between the PPC and PC in clear images that easily distinguish the dehiscence of the PC. This technique, in turn, improves the ability of the practitioner to choose the appropriate treatment option and to thereby avoid complications during cataract surgery.

## Figures and Tables

**Table 1:** Patient clinical data.

Demographics	
Age (years, mean ± SD)	63.64 ± 14.75
Male/female (*n*)	9/4
Right/left (*n*)	11/11
25 MHzB findings in the posterior capsule, *n* (%)	
Intact	19 (90.48%)
Dehiscence	2 (9.52%)
Intraoperative posterior capsule, *n* (%)	
Intact	17 (80.95%)
Ruptured	4 (19.05%)

**Table 2:** Status of PC in clinically diagnosed PPC.

	Slit lamp microscopy examination	25 MHzB	PC status during surgery
Case 1	PPC OU	Normal CPCC with minimal reflectivity between PC and PPC	Intact
Case 2	PPC OU	Normal CPCC with minimal reflectivity between PC and PPC	Intact
Case 3	PPC OD	Normal CPCC with minimal reflectivity between PC and PPC	Intact
Case 4	PPC OU	Normal CPCC with high reflectivity between PC and PPC	Intact with synechia
Case 5	PPC OU	Normal CPCC with high reflectivity between PC and PPC	Intact with synechia in the right eye and PCR in the left eye
Case 6	PPC OS	Normal CPCC with high reflectivity between PC and PPC	PCR
Case 7	PPC OU	Normal CPCC with minimal reflectivity between PC and PPC	Intact
Case 8	PPC OU	Normal CPCC with minimal reflectivity between PC and PPC	Intact
Case 9	PPC OS	Normal CPCC with minimal reflectivity between PC and PPC	Intact
Case 10	PPC OD	Normal CPCC with minimal reflectivity between PC and PPC	Intact
Case 11	PPC OU	Normal CPCC with minimal reflectivity between PC and PPC	Intact with synechia
Case 12	PPC OD	PC dehiscence, with reflectivity protruding to vitreous cavity	PCR with synechia
Case 13	PPC OD	PC dehiscence, with reflectivity protruding to vitreous cavity	PCR with synechia
Case 14	PPC OS	Normal CPCC with high reflectivity between PC and PPC	Intact with synechia
